# Dietary polyphenols and nutritional modulators in glioblastoma: mechanistic promise and translational barriers

**DOI:** 10.3389/fnut.2026.1902198

**Published:** 2026-09-14

**Authors:** Saniyah Shaikh, Arshiya Akbar, Ismail A. Abdullah, Shamsa Hilal Saleh, Jahan Salma, Mohammed Imran Khan, Ahmed Yaqinuddin, Itika Arora

**Affiliations:** 1 College of Medicine, Alfaisal University, Riyadh, Saudi Arabia; 2 King Faisal Specialist Hospital and Research Center (KFSHRC), Riyadh, Saudi Arabia; 3 King Faisal Specialist Hospital and Research Center (KFSHRC), Jeddah, Saudi Arabia

**Keywords:** curcumin, dietary polyphenols, EGCG, glioblastoma, ketogenic diet, omega-3 fatty acids, resveratrol, vitamin D

## Abstract

Glioblastoma is the deadliest primary brain tumor, and fewer than 7% of patients survive 5 years even after surgery, radiation, and chemotherapy. Several features make it difficult to treat: the tumor heterogeneity, it adapts quickly to resist drugs, and the blood-brain barrier keeps most agents from reaching it. These obstacles have renewed interest in adjuncts to standard care. Dietary polyphenols and certain nutritional compounds are among the candidates, since they can influence oxidative stress, inflammation, tumor metabolism, and the glioblastoma stem cells that drive recurrence. This review weighs the mechanistic, preclinical, and early clinical evidence for major polyphenols (curcumin, resveratrol, EGCG, quercetin, apigenin), non-polyphenol agents (omega-3 fatty acids, vitamin D, melatonin), and dietary patterns such as ketogenic, anti-inflammatory, and caloric restriction approaches. Across models these agents act on familiar oncogenic and stress pathways, including NF-κB, STAT3, PI3K/AKT/mTOR, and MAPK, curbing proliferation and invasion, triggering apoptosis, and sensitizing tumors to temozolomide and radiation. What the laboratory shows has rarely reached patients. Poor oral absorption, inconsistent brain penetration, heavy first-pass metabolism, individual variability, and a shortage of biomarker-guided trials stand in the way. We do not propose these agents as substitutes for treatment. Instead, we connect their biological promise to the pharmacokinetic, delivery, and trial design problems that decide whether they can ever help, treating them strictly as experimental adjuncts. By covering polyphenols, other nutritional agents, and whole dietary patterns together, the review offers a translational picture that earlier reviews have missed.

## 1 Introduction

Glioblastoma (GBM) is the most common and lethal primary malignant brain tumor in adults, accounting for approximately 50% of malignant central nervous system neoplasms and contributing disproportionately to cancer-related mortality and years of life lost (1). Despite decades of incremental therapeutic advances, prognosis remains poor, with contemporary series reporting 5-year relative survival rates below 7% and median overall survival rarely exceeding 18–20 months, even with maximal therapy (1, 2). This outcome reflects multiple intersecting biological and clinical challenges unique to GBM. The current standard of care, maximal safe resection followed by fractionated radiotherapy with concomitant and adjuvant temozolomide, was established two decades ago by the landmark Stupp trial and remains the backbone of first-line management (3). Tumor-treating fields, when added to maintenance temozolomide, provide a modest but statistically significant survival advantage and are increasingly incorporated into clinical practice (4). Anti-angiogenic therapy with bevacizumab improves radiographic responses and progression-free survival but has failed to demonstrate consistent overall survival benefit, and recurrence remains nearly universal and uniformly fatal (2, 5, 6). These outcomes stem from GBM's profound biological complexity. The disease exhibits marked inter- and intra-tumoral heterogeneity, characterized by coexisting transcriptional programs and dynamic cell-state plasticity that undermine single-target therapeutic approaches (6–8). Subpopulations of glioblastoma stem cells (GSCs) drive diffuse infiltration, immune evasion, metabolic adaptability, and resistance to radiotherapy and chemotherapy (7–9).

Compounding these cellular challenges, the blood–brain and blood–tumor barriers (BBB/BTB) severely restrict the penetration by most conventional and experimental agents into the central nervous system. The majority of small molecules and essentially all large biologics fail to achieve therapeutically relevant intraparenchymal concentrations, fundamentally limiting the translational applicability of systemic therapies developed for other cancers (10). Collectively, these features underscore the need for therapeutic strategies that extend beyond single-target cytotoxic approaches (2, 6). Within this context, systemic and host-directed modulators targeting metabolic, inflammatory, and immunologic axes have attracted increasing interest as potential adjuncts to standard therapy. Epidemiologic and mechanistic evidence suggests that dietary patterns and bioactive food components influence glioma biology through metabolic and inflammatory pathways (11). Among metabolic interventions, ketogenic and related low-carbohydrate diets aim to exploit GBM metabolic inflexibility, although robust evidence from randomized trials remains lacking (12, 13).

Against this broader nutritional backdrop, dietary polyphenols have emerged as exciting adjunctive candidates. Polyphenols, including curcumin, resveratrol, epigallocatechin-3-gallate (EGCG), quercetin, and apigenin, are widely distributed in plant-based foods and exert pleiotropic biological effects relevant to cancer biology, including modulation of redox homeostasis, inflammatory signaling, cell-cycle regulation, apoptosis, angiogenesis, and epigenetic mechanisms (14–17). Many of these effects converge on key signaling pathways, including NF-κB, STAT3, PI3K/AKT, and MAPK, which are critically dysregulated in GBM (16, 17). Although many polyphenols exhibit low oral bioavailability and extensive first-pass metabolism, mechanistic studies in oncology and neurobiology have delineated their molecular targets and identified formulation-dependent strategies to improve pharmacokinetic profiles (14, 16, 17). In preclinical GBM models, polyphenols inhibit tumor growth and invasion, suppress GSC self-renewal, and sensitize tumor cells to radiotherapy and temozolomide (15, 16, 18). Such pleiotropic, multi-targeted actions are conceptually attractive in a disease characterized by redundant and adaptive signaling networks (6, 15, 18). However, translation into clinically meaningful interventions remains constrained by pharmacokinetic and delivery barriers and, most critically, a paucity of rigorous GBM-specific clinical data (2, 10, 14, 18–21).

Given the widespread availability of polyphenol-rich foods, increasing use of over-the-counter nutraceuticals, and strong patient interest in diet-based adjuncts, a critical appraisal of the evidence base is essential (11, 15, 18). The primary contribution of this review is a translational synthesis that explains why the promising mechanistic activity of nutritional agents, when considered as adjuncts rather than alternatives to standard GBM therapy, has failed to translate clinically, and how future studies must address delivery, pharmacokinetics, and patient selection. Nutritional interventions are discussed strictly as experimental adjuncts, not as therapeutic alternatives. Several recent reviews have addressed polyphenols in GBM, including Beylerli et al. (18), Wong et al. (22), Smołińska et al. (23), and Justyńska et al. (24). The present review is distinguished from these prior works in three respects. First, its scope extends beyond polyphenols to encompass non-polyphenol nutritional agents with established central nervous system bioavailability—specifically omega-3 fatty acids, vitamin D, and melatonin—whose mechanisms of action in GBM are distinct and complementary. Second, it integrates evidence on whole dietary pattern strategies, including ketogenic, anti-inflammatory, and caloric restriction-based approaches, which operate through metabolic axes not addressed in polyphenol-focused reviews. Third, and most importantly, this review adopts an explicitly translational framing: rather than cataloging preclinical activity, it interrogates why mechanistic promise has consistently failed to translate into clinical benefit and identifies the pharmacokinetic, delivery, inter-individual, and trial design barriers that must be addressed before any nutritional intervention can be responsibly incorporated into GBM management. This integrated, critically appraisive scope constitutes the primary value added by the present synthesis.

To make the distinct contribution of this review explicit, and to address directly the legitimate concern that an expansion of scope alone does not constitute novelty, we compare our synthesis with the most closely related recent reviews in Table 1. Prior polyphenol-focused reviews (18, 22–24) primarily catalog mechanistic and preclinical activity, largely within a single compound class. The specific, and to our knowledge previously unaddressed, contribution of the present work is fourfold: (i) it integrates dietary polyphenols, non-polyphenol nutritional agents, and whole dietary patterns within a single translational framework; (ii) it applies a transparent, reproducible literature-selection strategy (see Search Strategy and Study Selection); (iii) it synthesizes the human and clinical evidence in a dedicated evidence table (Table 2) rather than restating preclinical findings; and (iv) it connects these interventions to emerging dimensions of glioblastoma biology-ferroptosis, immunometabolism, the myeloid compartment, extracellular vesicles, spatial metabolic heterogeneity, and the gut-brain axis-that are actively reshaping how metabolic and nutritional vulnerabilities are conceptualized. We therefore position this review not as a claim of mechanistic novelty, but as a critical, transparently sourced appraisal whose value lies in integrated synthesis and an honest account of why mechanistic promise has not translated into clinical benefit.

**Table 1:** Comparison of the present review with recent related reviews on natural compounds and nutrition in glioblastoma.

Feature	Beylerli (18)	Wong (22)	Smolinska (23)	Justynska (24)	Present review
Primary scope	Natural polyphenols in GBM	Flavonoids in high-grade glioma	Resveratrol & curcumin in glioma	Luteolin, quercetin, apigenin in glioma	Polyphenols + non-polyphenol agents + dietary patterns in GBM
Polyphenols covered	Multiple	Flavonoids only	Two compounds	Three flavonoids	Multiple + emerging (mangiferin, silymarin)
Non-polyphenol nutritional agents (omega-3, vitamin D, melatonin)	No	No	No	No	Yes
Whole dietary patterns (KD, Mediterranean, fasting)	No	No	No	No	Yes
Transparent search strategy/selection criteria	No	No	No	No	Yes
Dedicated synthesis of human/clinical evidence (table)	No	No	No	No	Yes
Explicit PK/BBB/delivery/trial-design focus	Partial	Partial	Partial	Partial	Central framing
Link to emerging TME biology (ferroptosis, immunometabolism, TAMs, EVs, spatial omics, gut-brain)	No	No	No	No	Yes
Practice-oriented recommendations	No	No	Partial	Partial	Yes

**Table 2:** Synthesis of human and clinical evidence for nutritional interventions in glioblastoma.

Intervention	Study (ref)	Design	*N*	Regimen	Endpoints	Key findings	Evidence level
Curcumin (micellar)	Dutzmann 2016 (30)	Single-arm feasibility	13 enrolled/10 completed	Oral micellar curcuminoids pre-surgery	Intratumoral concentration; 31P-MRS metabolism; safety	Quantifiable intratumoral curcumin (mean 56 pg/mg), sub-cytotoxic; metabolic shifts; no significant toxicity	Phase 0/feasibility
Polyphenol blend (polydatin, curcumin, Boswellia)	Bonucci 2025 (129)	Longitudinal pilot, non-randomized (integrative oncology added to Stupp)	72 (60 integrative/12 not)	Oral integrative therapy alongside standard care	Overall survival; safety	Longer median OS in adherent group (25.4 vs 10.6 mo); no severe AEs. Caveats: self-selected, tiny control, incomplete IDH data	Observational pilot (non-randomized)
Ketogenic diet	ERGO, Rieger 2014 (69)	Single-arm pilot feasibility (recurrent GBM)	20	Ketogenic diet	Feasibility; safety; PFS	Feasible and safe; limited monotherapy activity; supports use as an enhancer	Pilot/feasibility
Ketogenic metabolic therapy	Kiryttopoulos 2025 (13)	Single-center clinical study	Small cohort	Ketogenic diet + standard care	Adherence; survival signals	Improved outcomes with sustained ketosis; limited by no randomization/selection bias	Uncontrolled clinical
Ketogenic diet vs standard diet	DIET2TREAT (71, 72)	Randomized phase II (newly diagnosed GBM)	Planned RCT	KD vs standard diet + chemoradiation	Survival; QoL	Ongoing; will provide first randomized efficacy data	Ongoing phase II RCT
Melatonin + radiotherapy	Lissoni 1996 (65)	Randomized (RT +/- melatonin)	Small	20 mg/day melatonin + RT	Survival; tolerability	Improved 1-year survival; good tolerability. Pre-temozolomide era; RT-alone comparator now outdated	Historical RCT (pre-TMZ era)
Vitamin D	Frances 2017; Mulpur 2015 (57, 58)	Observational/retrospective	Cohorts	Baseline levels/supplement use	Survival association	Higher vitamin D associated with prolonged survival/reduced mortality; non-randomized, confounded	Observational
Omega-3 PUFAs (EPA/DHA)	Silva & Colquhoun 2025 (51)	Preclinical only	-	-	-	TMZ sensitization in GBM models; no controlled GBM trials exist	Preclinical only
Fasting/fasting-mimicking diet	Voss 2021; De Braud 2023 (80–82)	Pilot/phase II (feasibility)	Small	Cyclic FMD/short-term fasting during chemoradiation	Feasibility; tolerability; QoL	Feasible and generally safe; survival undefined	Pilot/feasibility

## 2 Search strategy and study selection

Although this article is a narrative rather than a systematic review, we followed a structured and transparent process for identifying and selecting the literature, consistent with current recommendations for improving the rigor and reproducibility of narrative reviews. We searched PubMed/MEDLINE, Scopus, and Web of Science from database inception to June 2026 using combinations of the terms “glioblastoma” OR “glioma” AND (“polyphenol”, “curcumin”, “resveratrol”, “EGCG/epigallocatechin”, “quercetin”, “apigenin”, “flavonoid”, “omega-3/docosahexaenoic acid”, “vitamin D”, “melatonin”, “ketogenic diet”, “caloric restriction”, “fasting”, “Mediterranean diet”, “nutraceutical”, “bioavailability”, “blood-brain barrier”, “nanoparticle”). Reference lists of retrieved articles and recent reviews were hand-searched to identify additional sources. We prioritized (i) mechanistic and preclinical studies in glioma/GBM models, (ii) all identified human studies and clinical trials of the relevant agents in GBM, and (iii) studies addressing pharmacokinetics, CNS delivery, and trial design. Peer-reviewed work from the last five years (2020-2026) was prioritized, while seminal earlier studies were retained where foundational. Agents were selected for in-depth discussion on the basis of (a) availability of GBM- or glioma-specific mechanistic data, (b) demonstrated or plausible CNS bioavailability, and (c) the existence of at least early human or robust preclinical evidence; compounds supported only by isolated *in vitro* reports in non-CNS systems were noted but not reviewed in depth. Conference abstracts without accessible data were included only where they represented the sole available evidence for an ongoing trial. We acknowledge that a narrative approach remains susceptible to selection bias; the criteria above are provided so that readers can appraise the completeness and balance of the evidence base.

### 2.1 Dietary polyphenols in GBM

Dietary polyphenols are plant-derived compounds that include phenolic acids, stilbenes, and several flavonoid subclasses such as flavonols, flavones, flavan-3-ols, and isoflavones. More than 8,000 polyphenolic structures have been identified, and their chemical diversity underlies broad anticancer activity by modulating oxidative stress, inflammation, cell-cycle regulation, apoptosis, angiogenesis, and invasion (25). In GBM, multiple polyphenols converge on a limited set of dysregulated oncogenic pathways, including NF-κB, STAT3, PI3K/AKT/mTOR, MAPK, and p53, which collectively drive tumor growth, invasion, stemness, immune evasion, and therapy resistance (25). NF-κB and STAT3 function as central transcriptional regulators of inflammatory signaling, cell survival, immune suppression, and the maintenance of glioma stem cells (GSCs). PI3K/AKT/mTOR integrates growth-factor signaling with metabolic adaptation, DNA damage tolerance, and redox homeostasis. Parallel activation of MAPK pathways further supports proliferation, stress adaptation, and invasive behavior, with extensive cross-talk amplifying pro-survival signaling outputs (16, 17, 25). Frequent p53 mutations or functional attenuation further permit unchecked cell-cycle progression in GBM (16, 17, 23).

The redundancy and co-activation of these pathways help explain why pleiotropic agents such as dietary polyphenols demonstrate broad preclinical activity in GBM models. This section focuses on the most extensively studied polyphenols in GBM—curcumin and resveratrol (non-flavonoids), and epigallocatechin-3-gallate (EGCG), quercetin, and apigenin (flavonoids). Other compounds, including luteolin, kaempferol, baicalein, and methoxyflavones, also exhibit anti-glioma activity but are supported by less extensive evidence (25). Figure 1 summarizes the principal oncogenic and metabolic pathways in GBM and highlights key nodes modulated by dietary polyphenols.

**Figure 1:**
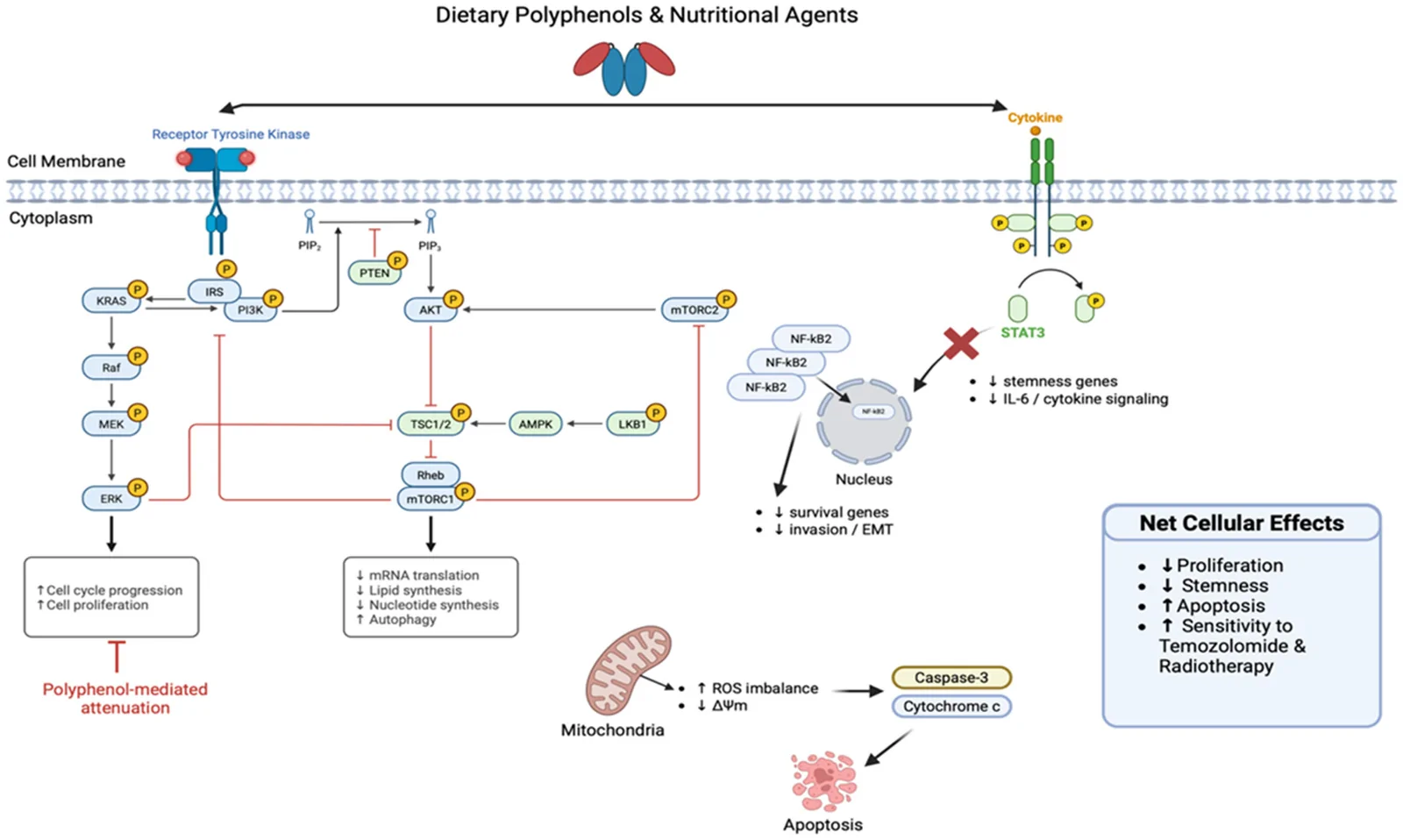
Molecular mechanisms by which dietary polyphenols disrupt oncogenic signaling and metabolic stress responses in glioblastoma. Schematic representation of the principal intracellular signaling pathways modulated by dietary polyphenols and selected nutritional agents in glioblastoma cells. These compounds attenuate receptor-mediated activation of the PI3K/AKT/mTOR and MAPK cascades and suppress cytokine-driven JAK/STAT3 signaling, leading to reduced cell-cycle progression, diminished stemness-associated transcriptional programs, and inhibition of inflammatory signaling. Concurrent inhibition of NF-κB nuclear activity further limits pro-survival gene expression and invasive or EMT-like phenotypes. In parallel, polyphenols perturb mitochondrial redox homeostasis, leading to increased reactive oxygen species (ROS), loss of mitochondrial membrane potential (ΔΨm), cytochrome c release, and activation of the caspase cascade. Collectively, these effects promote apoptotic cell death, impair glioma stem cell maintenance, and enhance sensitivity to standard therapies, including temozolomide and radiotherapy.

To avoid the redundancy inherent in re-describing a shared set of pathways for every compound, these canonical nodes (NF-kB, STAT3, PI3K/AKT/mTOR, MAPK, p53) are summarized once above. In the compound-specific sections that follow, we deliberately emphasize the distinctive molecular actions, tumor selectivity, and resistance-modifying properties that differentiate each agent, rather than restating the common signaling axis.

### 2.2 Curcumin

Curcumin, the principal bioactive compound in turmeric, is among the most extensively studied dietary polyphenols in GBM models. It suppresses convergent pro-survival and inflammatory signaling pathways central to GBM pathobiology, including NF-κB, STAT3, and PI3K/AKT/mTOR signaling (26–28). Curcumin additionally influences epigenetic regulation, leading to reduced GSC populations, impaired tumor sphere formation, and downregulation of stemness markers such as CD133 and Nestin (26, 27). In combination with temozolomide, curcumin demonstrates synergistic cytotoxicity and enhances radiosensitivity by increasing DNA damage and impairing repair mechanisms in GBM cells (28, 29). Notably, a feasibility study in GBM patients (n=13 enrolled, 10 completed) demonstrated that orally administered micellar curcuminoids achieved quantifiable intratumoral concentrations (mean curcumin 56 pg/mg tissue) and produced metabolic changes on ^3^^1^P magnetic resonance spectroscopic imaging, including increased intratumoral inorganic phosphate and a trend toward elevated intratumoral pH, without significant toxicity (30). Importantly, the intratumoral concentrations achieved were below those required for cytotoxic activity *in vitro*, underscoring the persistent pharmacokinetic challenge. Although nanoparticle-based formulations substantially improve CNS delivery and antitumor efficacy in preclinical models, clinical data remain limited (26, 27).

Distinctively, curcumin's most differentiated activity in GBM is epigenetic and stemness-directed - reducing CD133+/Nestin+ glioblastoma stem-cell populations and tumorsphere formation - rather than the shared pro-survival signaling it holds in common with other polyphenols (26, 27).

### 2.3 Resveratrol

Resveratrol, a stilbene found in grapes, berries, and peanuts, exerts multi-target anti-glioma effects with pronounced activity against therapy-resistant GSCs (31–33). Its mechanisms include modulation of NF-κB, STAT3, and PI3K/AKT signaling, as well as activation of tumor suppressor pathways such as p53 (31–33). Preclinical studies demonstrate that resveratrol enhances temozolomide efficacy by increasing DNA damage, reducing MGMT expression, and promoting GSC differentiation into less tumorigenic phenotypes (32). Resveratrol also attenuates inflammatory signaling within the tumor microenvironment (31). Despite these promising effects, clinical translation is hindered by poor aqueous solubility and rapid systemic metabolism. Nanoparticle-based delivery systems improve brain penetration and antitumor activity in animal models, but GBM-specific human data are lacking (31).

What most distinguishes resveratrol is its modulation of the Notch axis together with its capacity to lower MGMT expression, providing a specific mechanistic rationale for temozolomide re-sensitization that goes beyond generic pathway inhibition (32, 33).

### 2.4 Epigallocatechin-3-gallate (EGCG)

EGCG, the primary catechin in green tea, targets core GBM survival and stress-response pathways, including NF-κB, STAT3, and PI3K/AKT signaling (25, 34, 35). Notably, EGCG reverses chemotherapy-induced adaptive stress responses and drug efflux mechanisms. In orthotopic GBM models, EGCG alone has a limited impact on survival but significantly enhances temozolomide efficacy when combined (34). Mechanistically, EGCG inhibits temozolomide-induced endoplasmic reticulum stress responses, including GRP78 upregulation, while promoting apoptotic signaling (34). EGCG also downregulates P-glycoprotein, reducing drug efflux and increasing intracellular accumulation of chemotherapeutic agents (35). Combination strategies incorporating EGCG with temozolomide and metabolic modulators such as metformin further enhance cytotoxicity and disrupt tumor metabolic programs in preclinical studies (36, 37), highlighting EGCG's capacity to target multiple resistance mechanisms simultaneously.

EGCG's differentiating feature is direct antagonism of adaptive resistance machinery - downregulating P-glycoprotein-mediated efflux and suppressing temozolomide-induced GRP78/ER-stress cytoprotection - positioning it primarily as a resistance-reversing partner rather than a standalone cytotoxic agent (34, 35).

### 2.5 Quercetin and apigenin

Quercetin and apigenin are low-toxicity flavonoids with selective anti-glioma activity (25). Both compounds modulate GBM cell survival and stemness through upstream regulation of STAT3-dependent signaling, resulting in reduced invasion, enforced differentiation, and selective cytotoxicity toward tumor cells (38–40). Quercetin demonstrates notable tumor selectivity, inducing apoptosis in GBM cells while largely sparing normal astrocytes, and shows synergistic activity with temozolomide in preclinical models (38). Nanoparticle formulations further enhance their brain delivery and antitumor efficacy (25).

Apigenin induces G2/M cell-cycle arrest, promotes autophagy followed by apoptosis, and suppresses tumor growth in orthotopic GBM models while modulating immune-related components of the tumor microenvironment (40, 41).

Mechanistically, quercetin is set apart by pronounced tumor selectivity (sparing normal astrocytes) and suppression of GSK-3b/b-catenin/ZEB1-driven epithelial-mesenchymal-like invasion, whereas apigenin is distinguished by Cdk-cyclin-mediated G2/M arrest coupled to an autophagy-to-apoptosis switch (38–41).

### 2.6 Summary and Translational Context

Collectively, curcumin, resveratrol, EGCG, quercetin, and apigenin exert pleiotropic effects on GBM proliferation, invasion, GSC maintenance, and the tumor microenvironment. Their relative tumor selectivity and consistent synergism with temozolomide and radiotherapy make them attractive candidates for combination strategies (25, 38–41). However, translation into clinical benefit remains constrained by three significant barriers: poor oral bioavailability, limited blood–brain barrier penetration, and a lack of adequately powered GBM-specific clinical trials. Progress will require pharmaceutical-grade formulations, rigorous dose-finding and pharmacokinetic studies, and clinical trials demonstrating intratumoral target engagement (23, 24, 26, 27, 30, 31).

### 2.7 Emerging natural compounds beyond the classical polyphenols

Beyond the extensively studied agents above, recent preclinical work has begun to widen the natural-compound repertoire in GBM; these newer candidates both reinforce and usefully complicate the translational picture. Mangiferin, a xanthone polyphenol, was shown to target the glycolytic regulator PFKFB3, thereby suppressing PI3K/AKT/mTOR signaling, inducing S-phase arrest and apoptosis, and reducing tumor growth *in vivo* without overt systemic toxicity (42). This is notable because it couples a natural compound directly to a discrete, druggable metabolic node rather than to the diffuse pro-survival pathways typical of earlier polyphenols; nonetheless, mangiferin shares the low oral bioavailability that has constrained its predecessors, and no GBM- specific pharmacokinetic or clinical data yet exist. The flavonolignan silymarin has been reformulated within a lactoferrin-functionalized, brain-targeted nanoplatform that couples photodynamic reactive-oxygen-species generation and glycolytic suppression to PD-L1 degradation, improving both blood-brain barrier penetration and antitumor immunity in preclinical models (43). This exemplifies the field's shift from asking whether a compound is active to engineering how it reaches and reprograms the tumor, although such multicomponent nanosystems raise the manufacturing and regulatory hurdles discussed in the Limitations section. Finally, a standardized medicinal-mushroom blend combined with a fourth-generation platinum (IV)-perillyl alcohol prodrug produced greater cytotoxicity than either agent alone and activated multiple death modalities (apoptosis, mitophagy, and regulated necrosis) in GBM cells (44). Collectively, these studies broaden the mechanistic palette—glycolytic targeting, engineered delivery, and multimodal cell death - yet each remains at an early, exclusively preclinical stage and inherits the bioavailability, standardization, and trial-design barriers that are the central theme of this review.

## 3 Non-polyphenol nutritional compounds

Beyond dietary polyphenols, several non-polyphenol nutritional agents have attracted interest as potential adjuncts in glioblastoma (GBM), most notably omega-3 polyunsaturated fatty acids, vitamin D, and melatonin. These compounds are of interest because they modulate inflammation, lipid and cholesterol metabolism, cellular stress responses, and immune signaling through mechanisms distinct from classical cytotoxic agents and generally exhibit favorable safety profiles at nutritional doses. However, as with polyphenols, most supporting evidence derives from preclinical studies employing supraphysiologic exposures, and optimal dosing, formulation, and clinical benefit in GBM patients remain undefined.

### 3.1 Omega-3 fatty acids

Omega-3 polyunsaturated fatty acids (PUFAs), primarily eicosapentaenoic acid (EPA) and docosahexaenoic acid (DHA), are essential dietary fats that accumulate in the brain, where DHA is a major structural component of neuronal membranes (45). Omega-3 PUFAs compete with arachidonic acid–derived lipids, shifting eicosanoid production toward less pro-inflammatory mediators and specialized pro-resolving lipid species (46, 47). This is particularly relevant in GBM, where altered lipid metabolism increasingly appears to support tumor growth, invasion, and therapeutic resistance (45, 48). Preclinical studies demonstrate that omega-3 PUFAs modulate GBM lipid composition, attenuate tumor-promoting inflammatory signaling, and enhance sensitivity to temozolomide, including in chemoresistant and stem-like cell populations (49–51). Mechanistically, DHA and EPA influence fatty acid uptake and storage, alter glucose and lactate utilization, disrupt survival signaling, and modulate ABC transporter activity, thereby increasing intracellular retention of chemotherapeutic agents (50, 51). Emerging evidence further suggests that omega-3 PUFAs can reduce GBM cell migration and invasion through FABP-dependent mechanisms, indicating effects beyond inflammation alone (45, 48).

Despite these promising findings, clinical translation remains limited. Novel delivery strategies, such as DHA-enriched liposomal formulations, improve tumor targeting and reduce inflammatory markers in preclinical models (52). Still, no controlled clinical trials have established optimal dosing, formulation, or therapeutic benefit of omega-3 supplementation in GBM patients.

### 3.2 Vitamin D

Vitamin D, particularly its active metabolite calcitriol, functions as a neurosteroid hormone and exerts biological effects through the vitamin D receptor (VDR), which is expressed in both glioma cells and immune cells (53, 54). In GBM models, vitamin D exhibits direct anti-tumor activity by inhibiting proliferation, inducing apoptosis, suppressing migration and invasion, and reducing stem-like properties through VDR-dependent modulation of growth and survival pathways (53). Recent evidence indicates that vitamin D3 selectively impairs the survival of patient-derived glioma cells relative to normal astrocytes by disrupting cholesterol homeostasis, a process essential for tumor growth and membrane integrity (55). In human GBM specimens, higher VDR expression correlates with improved overall survival, and experimental modulation of VDR alters tumor cell migration and viability, highlighting the functional relevance of this pathway (54). However, heterogeneity in VDR signaling and vitamin D responsiveness suggests that patient stratification will be necessary for therapeutic application (56).

Clinical evidence in GBM remains primarily observational. Higher baseline vitamin D levels have been associated with prolonged survival in patients receiving standard therapy, and retrospective analyses suggest reduced mortality among patients using vitamin D supplements (57, 58). Broader oncologic studies report modest reductions in cancer-related mortality with vitamin D supplementation, likely mediated through effects on tumor invasiveness and immune regulation (59). Nevertheless, causality has not been established in GBM, and clinical use is constrained by risks of hypercalcemia at high doses and the presence of vitamin D–resistant tumor phenotypes.

The biology of the vitamin D receptor (VDR) itself is increasingly recognized as central both to these effects and to the heterogeneity of response. VDR is a ligand-activated nuclear transcription factor that, upon binding calcitriol, heterodimerizes with the retinoid X receptor and regulates genes governing cell-cycle arrest (e.g., CDKN1A/p21, CDKN1B/p27), apoptosis, differentiation, and immune modulation; in glioma, higher tumoral VDR expression correlates with improved overall survival, whereas Notch-associated and epigenetically silenced states can render cells 1,25(OH)_2_D_3_-resistant (53– 56). This receptor-level variability—driven by VDR polymorphisms, expression level, and cross-talk with Notch and cholesterol-homeostasis pathways - implies that vitamin D responsiveness is unlikely to be uniform across patients, and argues strongly for VDR-status-based stratification in any future trial rather than unselected supplementation.

### 3.3 Melatonin

Melatonin is an endogenous indoleamine with potent antioxidant, anti-inflammatory, and chronobiotic properties and readily crosses the blood–brain barrier (60). In GBM models, melatonin exerts direct anti-tumor effects by inhibiting proliferation, migration, and invasion; suppressing angiogenic signaling; and modulating autophagy and mitochondrial metabolism through redox-sensitive pathways (61). Experimental studies demonstrate that melatonin disrupts metabolic flexibility in GBM stem-like cells, inducing intracellular pH and energetic stress that reduces survival and clonogenic capacity while promoting cell death (62, 63). Melatonin also restores sensitivity to temozolomide in resistant GBM models and enhances cytotoxicity by modulating oxidative stress and DNA damage responses (61, 64).

Beyond tumor-intrinsic effects, melatonin modulates the tumor microenvironment by reducing expression of adhesion molecules and chemokines involved in monocyte recruitment, as well as enhancing natural killer cell and T-cell activity, thereby supporting anti-tumor immune responses (60, 61). Early clinical and observational studies, though limited in size, suggest improved survival and good tolerability when melatonin is administered alongside radiotherapy and temozolomide, with oral dosing regimens up to 20 mg/day demonstrating acceptable safety profiles (60, 64, 65). While definitive evidence is lacking, melatonin's favorable pharmacologic properties and low toxicity support continued evaluation in early-phase clinical trials.

Melatonin's actions in GBM are further contextualized by its role as the principal output of the circadian system. Beyond receptor-independent antioxidant activity, melatonin signals through MT1/MT2 receptors and entrains peripheral clock-gene machinery (e.g., BMAL1, PER, CRY), and there is growing evidence that circadian disruption and clock-gene dysregulation influence glioma growth, stemness, and treatment response. Because glioblastoma cells and their microenvironment retain partially functional circadian circuitry, the timing of both melatonin administration and conventional therapy (chronotherapy) may modulate efficacy - an underexplored dimension that could help reconcile the variable results of earlier melatonin studies and that warrants explicit incorporation into the design of future temozolomide-based trials (60, 61, 66).

Overall, omega-3 fatty acids, vitamin D, and melatonin represent mechanistically distinct yet complementary nutritional strategies in GBM. Omega-3 PUFAs primarily target lipid metabolism and inflammatory signaling; vitamin D exerts direct tumor-suppressive and immunomodulatory effects through VDR signaling; and melatonin acts as a pleiotropic modulator of metabolic stress, redox balance, and the tumor microenvironment. Although all three agents demonstrate favorable safety profiles and brain accessibility, their integration into clinical practice will require optimized formulations, biomarker-guided patient selection, and well-designed trials demonstrating intratumoral target engagement and clinical benefit alongside standard GBM therapy. Collectively, these agents converge on dysregulated metabolic, inflammatory, and oncogenic pathways in GBM, as summarized in Figure 2 and Table 3.

**Figure 2:**
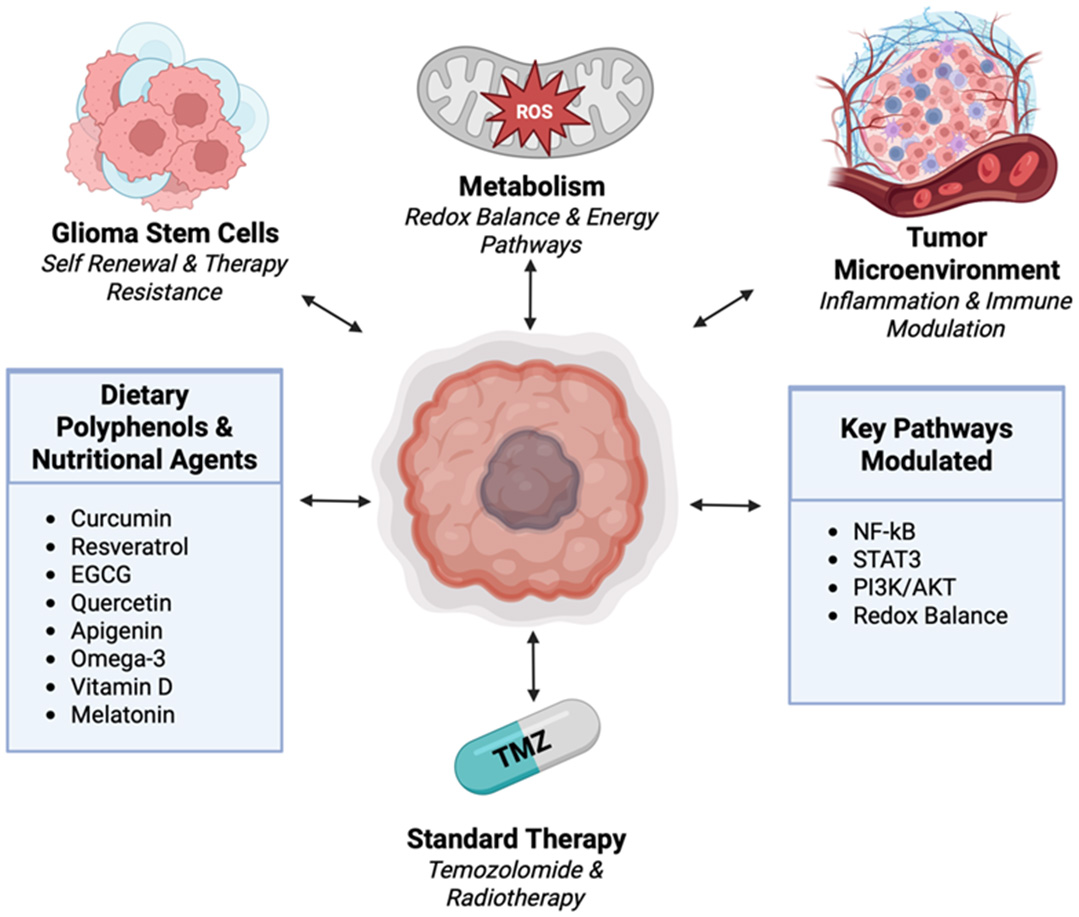
Nutritional modulation of glioblastoma biology. Schematic overview illustrating how dietary polyphenols and selected nutritional agents, including curcumin, resveratrol, epigallocatechin-3-gallate, quercetin, apigenin, omega-3 fatty acids, vitamin D, and melatonin, exert multi-target effects on glioblastoma biology. These agents influence multiple tumor-relevant compartments, including glioblastoma tumor cells, glioma stem cells, the tumor microenvironment, and cellular metabolism. Mechanistically, nutritional compounds modulate key oncogenic and stress-response pathways, including NF-κB, STAT3, PI3K/AKT signaling, and redox balance, thereby affecting proliferation, survival, inflammation, stemness, and metabolic adaptation. In addition, several agents enhance the sensitivity of preclinical models to standard therapies, including temozolomide and radiotherapy.

**Table 3:** Nutritional compounds in glioblastoma: mechanisms of action, blood–brain barrier penetration, and clinical evidence.

Compound	Class	Dietary sources	Key molecular targets	Anti-GBM mechanisms	BBB penetration	Clinical evidence in GBM	Main limitations	Refs
Curcumin	Curcuminoid	Turmeric	NF-κB, STAT3, PI3K/AKT/mTOR, COX-2, epigenetic regulators	Inhibits proliferation, induces apoptosis, reduces GSC stemness, sensitizes to TMZ/RT, anti-inflammatory	Low to modest (enhanced with formulation)	Micellar curcuminoids: feasibility study (*n* = 10) achieved intratumoral concentrations (mean 56 pg/mg tissue); concentrations were sub-cytotoxic	Very poor oral bioavailability, extensive first-pass metabolism, variable CNS penetration	(26–30)
Resveratrol	Stilbene	Grapes, berries, peanuts	PI3K/AKT/mTOR, STAT3, NF-κB, Notch, p53, MGMT	Induces apoptosis and autophagy, inhibits GSC self-renewal, enhances TMZ efficacy, anti-invasion	Moderate (improved with nanoformulations)	None in GBM; safety established in other cancers	Rapid metabolism, low aqueous solubility	(31–33)
EGCG	Catechin (flavan-3-ol)	Green tea	EGFR, PI3K/AKT, STAT3, ER stress (GRP78/CHOP), P-gp	Inhibits proliferation, potentiates TMZ, reduces GSC markers, reverses MDR	Low to moderate	No GBM trials; preclinical synergy with TMZ/metformin	Hepatotoxicity risk at high doses (>800 mg/day), instability	(25, 34–37)
Quercetin	Flavonol	Onions, apples, berries	Axl/IL-6/STAT3, GSK-3β/β-catenin/ZEB1, mitochondrial apoptosis	Induces apoptosis, suppresses invasion, reverses EMT-like phenotype, some tumor selectivity	Low (enhanced with nanocarriers)	None in GBM	Poor solubility, limited CNS accumulation	(25, 38, 39)
Apigenin	Flavone	Celery, parsley, chamomile	Cdk-cyclins, caspase-3, ROS pathways	Induces G2/M arrest, triggers autophagy and apoptosis, modulates tumor microenvironment	Modest	None in GBM	Preclinical data only, formulation challenges	(40, 41)
Omega-3 PUFAs (EPA/DHA)	Essential fatty acids	Fish, fish oil	Lipid metabolism, ABC transporters, inflammatory eicosanoids	Modulates lipid metabolism, reduces pro-inflammatory eicosanoids, sensitizes to TMZ	High (physiologic brain accumulation in neuronal membranes)	None; observational data in supportive care only	Theoretical bleeding risk at very high doses	(45–52)
Vitamin D (Calcitriol)	Neurosteroid	Endogenous synthesis, supplements	VDR-mediated transcription, cholesterol, immune pathways	Induces cell cycle arrest and apoptosis, suppresses cholesterol homeostasis, immune modulation	High (physiologic BBB penetration)	Observational associations; no RCTs in GBM	Hypercalcemia at high doses; some GBM cell lines resistant	(53–59)
Melatonin	Indoleamine	Endogenous; oral supplements	Mitochondrial metabolism, ROS, SIRT1, ICAM-1/CCL2	Inhibits proliferation/invasion, induces metabolic stress, modulates tumor microenvironment, enhances TMZ/RT	Excellent	Small trials (20 mg/day); possible survival benefit; pre-TMZ era data	Excellent safety; somnolence, vivid dreams	(60–65)

## 4 Dietary patterns and GBM

Individual nutrients and bioactive compounds act within the context of overall dietary patterns, which shape systemic metabolism, inflammatory tone, and hormonal signaling—factors that can meaningfully influence glioma biology. In glioblastoma (GBM), three dietary strategies have attracted particular attention: ketogenic and other low-carbohydrate diets, anti-inflammatory nutritional patterns such as Mediterranean or plant-based diets, and caloric restriction or fasting-mimicking approaches. Although evidence remains heterogeneous and predominantly early-stage, these strategies converge mechanistically on modulation of glucose and ketone availability, insulin/IGF-1 signaling, systemic inflammation, and oxidative stress (11, 12, 67, 68).

### 4.1 Ketogenic diets

The ketogenic diet (KD) is a high-fat, very low-carbohydrate, adequate-protein dietary pattern that induces sustained ketosis, characterized by elevated circulating ketone bodies and reduced glucose and insulin levels. Its rationale in GBM derives from the Warburg effect—the preferential reliance of cancer cells on glycolysis even in the presence of oxygen—and evidence that many glioma cells exhibit limited metabolic flexibility, relying heavily on glucose metabolism while demonstrating impaired ketone body utilization relative to normal neurons (12, 69). In preclinical glioma models, ketogenic diets slow tumor growth, reduce intratumoral lactate accumulation, suppress angiogenic signaling, and improve survival, particularly when combined with radiotherapy or chemotherapy (12, 69). These effects are associated with reduced insulin/IGF-1 signaling, attenuation of PI3K/AKT/mTOR and RAS pathway activation, increased oxidative stress in metabolically inflexible tumor cells, and modulation of the tumor immune microenvironment.

Clinical experience with KD in GBM is limited to small pilot and feasibility studies. In recurrent GBM, the ERGO trial a single-arm pilot feasibility study demonstrated that KD is feasible and generally safe but has limited activity as monotherapy, supporting its role as a potential treatment enhancer rather than a standalone intervention (69). Building on this platform, the subsequent ERGO2 trial evaluated regorafenib in relapsed or refractory GBM, demonstrating that the same patient population could be enrolled into sequential early-phase interventional studies and providing important feasibility data for designing further trials in this setting (70). In newly diagnosed disease, early-phase studies combining KD with standard chemoradiotherapy reported acceptable adherence, manageable toxicity, and stable or improved quality-of-life measures, with exploratory survival outcomes exceeding historical controls. However, these trials were not designed to assess efficacy (13, 69). A single-center study suggested improved outcomes among patients maintaining ketosis for prolonged periods, but interpretation is limited by a lack of randomization and potential selection bias (13). Ongoing randomized phase II trials, including DIET2TREAT, are evaluating the impact of KD on survival and quality of life (71, 72). Despite encouraging feasibility, implementation challenges such as weight loss, sarcopenia risk, dietary complexity, and protocol heterogeneity currently limit routine clinical adoption (12, 13).

### 4.2 Anti-inflammatory dietary patterns

Chronic systemic inflammation and metabolic dysfunction are increasingly implicated in glioma development and progression. Anti-inflammatory dietary patterns, particularly Mediterranean and whole-food plant-based diets, are rich in fruits, vegetables, whole grains, legumes, olive oil, fish, and bioactive compounds, while being low in processed foods and added sugars (11, 68). Epidemiologic studies consistently associate higher adherence to Mediterranean and healthy plant-based dietary patterns with reduced glioma risk (73–75). These diets are associated with lower systemic inflammation, improved insulin sensitivity, reduced IGF-1 signaling, and favorable gut microbiome profiles, providing mechanistic plausibility for modulation of glioma risk (11, 76). However, in patients with established GBM, no randomized trials have evaluated Mediterranean or plant-based diets as therapeutic interventions. Previous studies support the benefits of general health and supportive care during treatment, but survival advantages remain unproven (12, 68). Consequently, such dietary patterns are best viewed as foundational supportive strategies, provided adequate caloric and protein intake is maintained to prevent malnutrition.

### 4.3 Caloric restriction and fasting-mimicking diets

Caloric restriction (CR), intermittent fasting, and fasting-mimicking diets (FMDs) have been proposed as metabolic strategies to enhance treatment response by transiently lowering glucose, insulin, IGF-1, and leptin levels and inducing differential stress resistance. Under these conditions, normal cells activate protective programs, whereas cancer cells become more vulnerable to cytotoxic therapies (69, 77, 78).

In glioma models, short-term fasting markedly enhances the efficacy of chemotherapy and radiotherapy, increasing tumor DNA damage and apoptosis while protecting normal tissues, a phenomenon termed differential stress sensitization (78, 79). Early clinical studies suggest that short-term fasting or cyclic FMDs during chemoradiotherapy are feasible and generally safe, with manageable weight loss and potential improvements in fatigue and quality of life (80, 81). However, survival outcomes remain undefined, and these approaches carry nontrivial risks, including sarcopenia, metabolic disturbances, and contraindications in frail or nutritionally compromised patients. At present, CR and FMD strategies should be restricted to clinical trials or closely supervised protocols with rigorous patient selection and medical oversight (78, 82, 83).

### 4.4 Integrated perspective

Ketogenic diets, anti-inflammatory dietary patterns, and caloric restriction–based approaches represent distinct but potentially complementary strategies for modulating GBM biology. Ketogenic diets primarily target tumor metabolic inflexibility; Mediterranean and plant-based diets reduce systemic inflammation and provide sustained exposure to beneficial bioactive compounds; and fasting-mimicking strategies induce transient metabolic stress that may sensitize tumors to therapy. All three converge on attenuation of insulin/IGF-1 signaling, a pathway implicated in GBM growth and resistance.

Optimal implementation will likely require patient-specific selection, integrating nutritional status, tumor metabolic characteristics, and treatment context. Biomarker-driven trials incorporating metabolic imaging, circulating metabolites, and molecular tumor profiling will be essential to identify patients most likely to benefit from each approach (12, 68, 69, 77). The major dietary patterns evaluated in GBM, along with their evidence base and implementation considerations, are summarized in Table 4.

**Table 4:** Dietary patterns in glioblastoma: mechanistic rationale, clinical evidence, and implementation considerations.

Dietary pattern	Metabolic rationale	Preclinical evidence	Clinical evidence in GBM	Feasibility & safety	Patient selection	Current recommendation	Refs
Ketogenic diet (KD)	Exploits GBM glucose reliance; elevates ketones; lowers insulin/IGF-1	Slows tumor growth, reduces lactate, improves survival with RT/chemo in orthotopic models	Phase I trials: feasible and safe; exploratory survival signals; ongoing Phase II RCTs (DIET2TREAT)	Requires dietitian support; risk of weight loss/sarcopenia	Patients with adequate performance status and nutritional reserve	Investigational; await RCT results	(12, 13, 69–72)
Anti-Inflammatory (mediterranean/plant-based)	Reduces systemic inflammation; improves insulin sensitivity; rich in polyphenols and omega-3 s	Epidemiologic: higher adherence associated with lower glioma risk; mechanistic plausibility via CRP, IGF-1, microbiota	No intervention trials in established GBM	Highly feasible, culturally adaptable, safe	All patients, provided adequate energy/protein intake	Recommended as part of supportive care for general health benefits	(11, 68, 73–76)
Caloric restriction/fasting-mimicking diet (FMD)	Differential stress resistance; lowers glucose, IGF-1, leptin	Short-term fasting enhances chemo/RT efficacy; increases tumor apoptosis in glioma models	Pilot studies: feasible, safe, manageable weight loss; survival data pending	Requires medical supervision; contraindicated in frail or cachectic patients	Carefully selected patients with good nutritional status, no sarcopenia	Investigational; restrict to clinical trials or closely monitored protocols	(69, 77–82)

## 5 Emerging themes linking nutritional interventions to glioblastoma biology

Several rapidly evolving areas of glioblastoma biology are reframing how nutritional and natural-compound interventions might act. Integrating them clarifies both new opportunities and the limits of current evidence, and situates the agents discussed above within the mechanisms that now dominate the field. Ferroptosis. Iron-dependent, lipid-peroxidation-driven cell death has emerged as a targetable vulnerability in GBM, and several nutritional agents converge on it. Curcumin analogs disrupt GPX4-mediated redox homeostasis to sensitize temozolomide-resistant GBM to ferroptotic death (84), while dietary omega-3 polyunsaturated fatty acids, once incorporated into membrane phospholipids, undergo peroxidation in the acidic tumor microenvironment to trigger ferroptosis (85). This provides a coherent bridge between lipid-based nutritional interventions and a specific, druggable death pathway, although the pro-oxidant requirement sits in tension with the antioxidant framing of many polyphenols and must be reconciled in dose- and context-specific terms.

Immunometabolism. The GBM microenvironment enforces nutrient competition - depleting glucose, tryptophan, and arginine - to disable cytotoxic T cells (86), and metabolic-dietary interventions intersect directly with this axis. The ketogenic diet, while extending survival in models, can paradoxically favor immunosuppressive M2 macrophage programs unless combined with myeloid-targeted agents (87), illustrating that metabolic interventions reshape - rather than simply suppress - the immune microenvironment and may require rational immunometabolic combinations. Tumor-associated macrophages and microglia. Myeloid cells constitute 30-50% of the GBM mass and are a major nutritional target (88). Bioavailable curcumin formulations repolarize glioma-associated microglia/macrophages from a pro-tumor M2 toward an anti-tumor M1-like state and recruit NK-cell-dependent cytotoxicity (89), offering a plausible immunologic mechanism for polyphenols that is independent of direct tumor-cell killing.

Extracellular vesicles. EVs shape GBM progression by transferring immunosuppressive and pro-invasive cargo, and also represent a natural route across the blood-brain barrier (90, 91). Notably, exosomes released from curcumin- and/or temozolomide-treated GBM cells carry pro-apoptotic signals to naive tumor cells (92), suggesting that nutritional agents may act partly through modification of EV cargo - a mechanistically distinct and underexplored mode of action. Single-cell and spatial transcriptomics. These technologies have revealed that GBM comprises coexisting metabolic states - including glycolytic/plurimetabolic and mitochondrial/OXPHOS programs - with distinct therapeutic vulnerabilities and spatially variable nutrient access (93, 94). In-patient 13C-glucose tracing confirms cell-intrinsic, spatially distinct glucose-metabolism phenotypes (95), implying that metabolic and dietary interventions will engage only the subset of tumor regions matching their metabolic dependency, and that biomarker- and imaging-guided selection is essential. Gut-brain axis. The gut microbiome is increasingly implicated in glioma biology: gut microbiota shape anti-glioma innate immunity (96), and a ketogenic diet was recently shown to restrain glioma progression through microbiota-derived butyrate (97). Because the microbiome also governs the biotransformation and systemic exposure of dietary polyphenols, inter-individual microbiome variation is simultaneously a source of the pharmacokinetic variability discussed below and a potentially modifiable therapeutic lever (98).

## 6 Limitations and translational challenges

Strong preclinical evidence supporting dietary polyphenols and nutritional modulators in glioblastoma (GBM) contrasts sharply with limited and largely inconclusive clinical data. This translational gap reflects fundamental barriers that must be addressed before these agents can be meaningfully integrated into GBM management. These challenges arise along the entire pathway from oral intake to intratumoral exposure and clinical evaluation, as summarized schematically in Figure 3.

**Figure 3:**
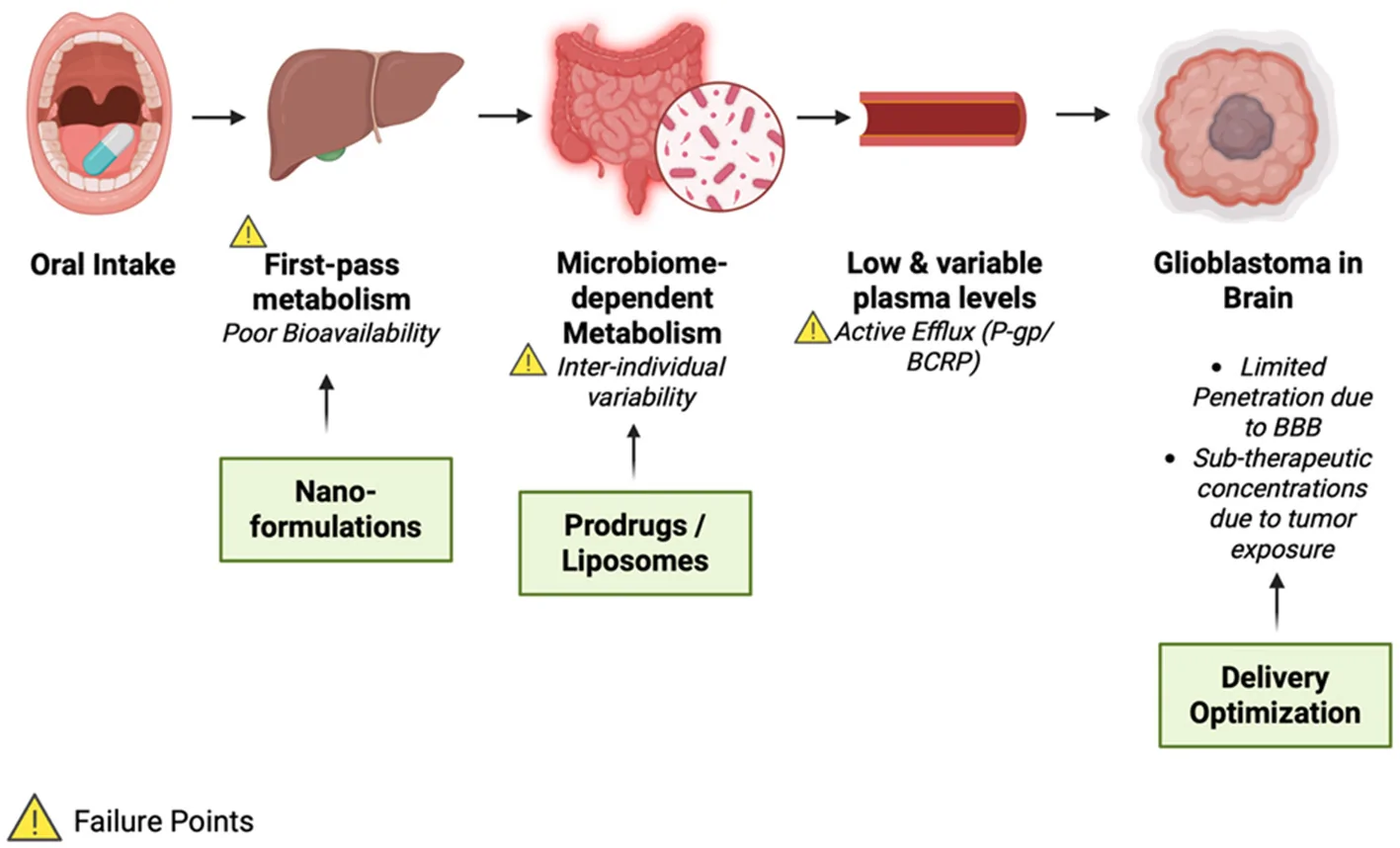
From diet to tumor: translational barriers limiting the efficacy of nutritional interventions in glioblastoma. Schematic representation of the sequential barriers encountered by dietary polyphenols and selected nutritional compounds from oral intake to glioblastoma tumor exposure. Following ingestion, compounds undergo extensive hepatic first-pass metabolism and microbiome-dependent biotransformation, resulting in poor, highly variable systemic bioavailability. Low and inconsistent plasma concentrations, together with active efflux transporters (e.g., P-glycoprotein and BCRP) and the restrictive properties of the blood–brain barrier, further limit central nervous system and intratumoral delivery, frequently leading to sub-therapeutic tumor exposure. Key failure points along the translational pathway are highlighted. Potential mitigation strategies, including nanoformulations, prodrugs, and liposomal approaches, are indicated as intervention points to enhance stability, bioavailability, brain penetration, and tumor exposure.

### 6.1 Bioavailability and blood–brain barrier constraints

A significant limitation of dietary polyphenols is their poor oral bioavailability, driven by low aqueous solubility, gastrointestinal instability, extensive hepatic first-pass metabolism, and rapid systemic clearance (83, 99). Consequently, plasma concentrations achieved with conventional dietary or supplement dosing are often orders of magnitude lower than those used in preclinical experiments, raising concerns regarding clinical relevance. Blood–brain barrier (BBB) penetration is an additional, often underestimated obstacle. Although certain polyphenols, including curcumin, resveratrol, and selected flavonoids, can cross the BBB via passive diffusion or transporter-mediated mechanisms, brain accumulation is generally modest and highly variable (99–102). Active efflux transporters, such as P-gp and BCRP, further limit central nervous system exposure, often resulting in intratumoral concentrations well below those required for biological activity *in vitro*.

To address these challenges, pharmaceutical strategies including nanoencapsulation, lipid- and polymer-based carriers, liposomes, micelles, prodrug approaches, and cyclodextrin complexes have been developed to enhance stability, systemic exposure, and brain delivery (103, 104). While these approaches substantially improve pharmacokinetic profiles and antitumor efficacy in animal models, human data in GBM remain sparse, limited to small exploratory studies (30). BBB penetration and target engagement remain largely unvalidated in clinical settings. Blood–brain barrier penetration challenges and formulation strategies in glioblastoma are summarized in Table 5.

**Table 5:** Blood–brain barrier penetration challenges and formulation strategies in glioblastoma.

Challenge	Compounds affected	Mechanism of limited penetration	Formulation solutions	Clinical translation status	Refs
Low aqueous solubility	Curcumin, resveratrol, quercetin	Poor dissolution in GI tract and plasma	Micelles, liposomes, solid lipid nanoparticles, inclusion complexes	Micellar curcumin: phase I data in GBM; others preclinical	(30, 83, 99–103)
Extensive first-pass metabolism	All polyphenols	Phase II conjugation (glucuronidation, sulfation, methylation)	Nanoencapsulation to protect from metabolism; metabolically stable analogs	Limited human data; mostly preclinical	(83, 99, 101, 104)
Active efflux (P-gp, BCRP)	EGCG, quercetin, curcumin	Transporter-mediated extrusion from brain	P-gp inhibitors; nanocarriers that bypass efflux	Preclinical; no clinical trials in GBM	(99–102)
Rapid systemic clearance	Resveratrol, EGCG	Short half-life, high plasma protein binding	Sustained-release formulations; nanoparticles with prolonged circulation	Phase I studies in other cancers; not GBM-specific	(103, 118, 119)

Two features further complicate CNS exposure and are often underappreciated. First, the blood-brain barrier in GBM is heterogeneously, not uniformly, disrupted: contrast enhancement marks only regions of gross barrier breakdown, whereas the infiltrative tumor margins responsible for recurrence lie behind a largely intact barrier, creating pharmacologic sanctuary sites where nutritional agents - like conventional drugs - are systematically under-dosed (105). Second, intratumoral pharmacokinetics vary spatially; mechanistic CNS pharmacokinetic modeling predicts markedly different drug exposure across the enhancing core, infiltrative rim, and normal-appearing brain (106), so a single plasma or bulk-tissue concentration poorly represents exposure at the cells that matter. These realities motivate delivery routes that bypass the barrier altogether, such as intranasal administration of lipophilic terpenes: intranasal perillyl alcohol and its conjugates (e.g., NEO212) exemplify a natural-compound strategy that achieves CNS delivery while circumventing first-pass metabolism, and provide a template against which nutritional formulations should be benchmarked (107). Where nanoparticle formulations are pursued to overcome these barriers, their clinical translation faces additional, frequently underestimated regulatory and manufacturing hurdles - inconsistent nanomaterial definitions, batch-to-batch reproducibility, scale-up, and evolving guidance - that must be addressed early through quality-by-design and regulatory engagement (108, 109). Collectively, these considerations argue that biomarker- and imaging-based patient selection - matching a given agent to tumors with the requisite metabolic dependency, barrier status, and receptor expression - is a prerequisite, not an afterthought, for credible trials.

### 6.2 Inter-individual variability in metabolism and exposure

Even with optimized formulations, substantial inter-individual variability complicates the translation of nutritional agents. Polyphenol metabolism is strongly influenced by gut microbiota composition, which determines the generation of bioactive metabolites and varies widely between individuals (101, 104, 110). Antibiotic exposure, dietary patterns, and baseline dysbiosis can markedly alter systemic and brain exposure. Genetic polymorphisms in phase II metabolic enzymes (e.g., UGT, SULT, COMT) and drug transporters (e.g., ABCB1/P-glycoprotein, ABCG2/BCRP) further influence absorption, clearance, and tissue distribution (83, 104). Physiological and environmental factors, including liver function, circadian variation, and concurrent medications, also affect first-pass metabolism. Of particular relevance in GBM, enzyme-inducing antiepileptic drugs may accelerate polyphenol clearance, reducing adequate exposure. Collectively, this variability complicates dose selection, obscures dose–response relationships, and suggests that uniform dosing strategies are unlikely to be optimal. Future clinical studies should incorporate pharmacokinetic monitoring, microbiome profiling, and genetic stratification to better account for inter-individual differences.

### 6.3 Safety, toxicity, and drug–nutrient interactions

Although generally safe at dietary levels, pharmacologic dosing of nutritional compounds introduces nontrivial safety considerations. Curcumin is typically well tolerated at high oral doses, with gastrointestinal adverse effects most commonly reported; however, rare cases of hepatotoxicity, drug–enzyme interactions, and contamination of commercial preparations have been described (111–113). Concentrated green tea extracts and high-dose EGCG (typically >799 mg/day in susceptible individuals) have been associated with liver injury, with both animal and human reports documenting hepatotoxicity at supraphysiologic exposures (114–117). Resveratrol is generally well tolerated at moderate doses but has produced unexpected systemic toxicity and renal effects at very high doses in oncology trials, leading to early termination in some settings (118, 119). Among non-polyphenol agents, vitamin D carries a risk of hypercalcemia and nephrocalcinosis at high doses, necessitating careful monitoring, while omega-3 polyunsaturated fatty acids may theoretically increase bleeding risk at very high doses. Melatonin exhibits a favorable safety profile even at relatively high doses, with sedation and vivid dreams as the most commonly reported effects, and appears well tolerated in combination with radiotherapy and chemotherapy (120–123).

Beyond intrinsic toxicity, drug–nutrient interactions represent a critical concern in GBM patients receiving complex multidrug regimens. Polyphenols such as curcumin, resveratrol, and EGCG can modulate cytochrome P450 enzymes and drug transporters, potentially altering exposure to chemotherapeutics, antiepileptics, and corticosteroids. Moreover, the antioxidant properties of some compounds raise theoretical concerns regarding interference with radiation- or chemotherapy-induced oxidative damage, underscoring the need for rigorous interaction and safety assessment. Importantly, “natural” origin does not equate to safety, and nutritional agents require the same level of pharmacologic scrutiny as conventional drugs.

### 6.4 Clinical trial design and evidence quality

Despite extensive preclinical investigation, clinical evidence supporting nutritional interventions in GBM remains limited. Among dietary polyphenols, only curcumin has been evaluated in a GBM-specific human study, demonstrating intratumoral exposure with a micellar formulation but without survival endpoints (30). Other polyphenols, including resveratrol, EGCG, quercetin, and apigenin, have not undergone completed therapeutic trials in GBM patients. Evidence for non-polyphenol agents is similarly sparse, with vitamin D and melatonin supported primarily by observational studies or small, older trials, and omega-3 fatty acids remaining confined to preclinical models. Methodological barriers further hinder progress, including a lack of standardized formulations with defined pharmacokinetics, the absence of GBM-specific dose-finding studies, limited assessment of intratumoral target engagement, substantial patient heterogeneity, and weak commercial incentives due to over-the-counter availability. Addressing these challenges will require coordinated efforts to develop pharmaceutical-grade formulations, implement adaptive trial designs incorporating pharmacokinetic and pharmacodynamic endpoints, stratify patients using molecular and metabolic biomarkers, and integrate nutritional agents into combination strategies with standard therapy from the outset.

The translational barriers facing nutritional interventions in GBM are substantial but not insurmountable. Poor bioavailability and BBB penetration can be addressed through advanced formulation strategies; inter-individual variability may be mitigated through personalized, biomarker-informed approaches; safety concerns require rigorous pharmacologic evaluation; and evidence gaps demand well-designed, adequately powered clinical trials. Progress will depend on applying the same methodological rigor to nutritional agents as to conventional therapeutics, rather than extrapolating clinical relevance from preclinical models or assuming safety based on dietary origin.

## 7 Translational and clinical perspective

### 7.1 Current state of clinical evidence

Despite extensive preclinical investigation, clinical evidence supporting dietary polyphenols and nutritional interventions in glioblastoma (GBM) remains limited and heterogeneous. Among polyphenols, only curcumin has been evaluated in a GBM-specific human study a feasibility study demonstrating that a micellar curcuminoid formulation can achieve quantifiable intratumoral concentrations and produce metabolic changes on ^3^^1^P MRS imaging, but at concentrations below those required for cytotoxic activity *in vitro* and without survival or efficacy endpoints (30). Other polyphenols, including resveratrol, EGCG, quercetin, and apigenin, lack completed therapeutic trials in GBM patients, with existing clinical data in other cancers showing acceptable safety but inconsistent efficacy (103, 124–126). Clinical data for non-polyphenol nutritional agents are similarly sparse. Observational studies suggest that higher baseline vitamin D levels are associated with prolonged survival in GBM patients receiving standard therapy, but these findings are non-randomized and potentially confounded (54, 57, 58). Small, older trials evaluating melatonin in combination with radiotherapy reported improved short-term survival and good tolerability; however, these trials were conducted before temozolomide became standard of care and used comparator arms (radiotherapy alone) that are no longer standard practice, limiting their interpretability (65, 66). For omega-3 polyunsaturated fatty acids, no controlled clinical trials in GBM exist, and evidence remains confined to preclinical models (51).

Evidence for dietary pattern interventions follows a similar trajectory. Ketogenic diets have demonstrated feasibility and acceptable safety in early-phase trials, with exploratory survival signals that require confirmation in randomized studies (12, 13, 69). Ongoing phase II trials, including DIET2TREAT, will be critical in determining clinical impact (71, 72). Data on fasting or fasting-mimicking diets are preliminary and limited mainly to feasibility and tolerability endpoints (80, 82). In contrast, epidemiologic studies support Mediterranean and plant-based diets but lack interventional data in established GBM (68, 73). Collectively, current evidence is characterized by strong mechanistic and preclinical support, limited pharmacokinetic validation in humans, and a near absence of robust efficacy data. At present, none of these interventions can be stated with confidence to improve survival, quality of life, or other clinically meaningful outcomes when added to standard GBM therapy. The available human and clinical evidence across these interventions is synthesized in Table 2.

### 7.2 Conceptual framework: nutritional agents as adjunctive therapy

Nutritional agents are best conceptualized as adjuncts to standard-of-care therapy rather than as stand-alone treatments. Preclinical data suggest three principal modes through which such interventions may exert benefit: enhancement of chemo- and radiosensitivity, metabolic reprogramming, and modulation of the tumor microenvironment. Polyphenols such as curcumin, resveratrol, EGCG, and melatonin enhance temozolomide- and radiation-induced cytotoxicity in GBM models by suppressing pro-survival signaling pathways, impairing DNA damage repair mechanisms, and targeting therapy-resistant glioblastoma stem cell populations (23, 31, 127, 128). Metabolic interventions, including ketogenic and fasting-mimicking diets, reduce glucose and insulin/IGF-1 signaling while increasing ketone availability, potentially exploiting GBM metabolic inflexibility. Omega-3 fatty acids and selected polyphenols further influence lipid metabolism and mitochondrial function, contributing to metabolic stress within tumor cells (12, 51).

In parallel, polyphenols, vitamin D, melatonin, and anti-inflammatory dietary patterns attenuate pro-tumor inflammatory signaling, modulate immune cell recruitment and function, and may partially counteract GBM-associated immunosuppression (54, 60, 61, 68, 73). These effects raise the possibility that nutritional strategies could complement emerging immunotherapeutic approaches, although this remains speculative and untested in clinical trials.

### 7.3 Future directions and research priorities

Meaningful clinical translation will require treating nutritional agents with the same rigor applied to conventional therapeutics. Priorities include development of pharmaceutical-grade, brain-penetrant formulations with defined human pharmacokinetics; early-phase trials incorporating cerebrospinal fluid and tumor tissue sampling to confirm intratumoral exposure and target engagement; and biomarker-driven patient selection based on metabolic, molecular, and immune features.

Future studies should evaluate nutritional agents in rational combination with standard therapies and emerging modalities, incorporate adaptive trial designs with pharmacodynamic endpoints, and prioritize patient-centered outcomes, including quality of life, cognitive function, and treatment tolerance. Without such rigor, promising preclinical findings are unlikely to translate into clinical benefit.

### 7.4 Clinical implications for current practice

Until definitive evidence becomes available, nutritional interventions in GBM should be approached conservatively. Whole-food dietary patterns with established general health benefits, such as Mediterranean or plant-based diets, are reasonable when caloric and protein requirements are met. Ketogenic and fasting-based strategies should be restricted to motivated patients within clinical trials or under specialized dietary supervision. Vitamin D deficiency should be screened and corrected according to standard guidelines. Melatonin may be considered a low-risk adjunct for sleep or quality-of-life support. High-dose polyphenol supplementation is not recommended outside clinical trials due to uncertain efficacy and potential drug interactions, while omega-3–rich foods are appropriate, but the supplemental benefit remains unproven.

The path from laboratory promise to clinical utility for nutritional interventions in GBM remains challenging. Progress will depend on pharmaceutical-grade formulation, rigorous pharmacokinetic and mechanistic validation, biomarker-informed patient selection, and adequately powered clinical trials with clinically meaningful endpoints. Until such data are available, recommendations should adhere to the same evidence standards applied to conventional cancer therapies rather than relying solely on preclinical findings.

## 8 Conclusion

This review critically evaluates dietary polyphenols and nutritional modulators with potential relevance to glioblastoma (GBM). Although extensive preclinical evidence demonstrates biological activity of these agents in GBM models, their clinical utility remains fundamentally constrained by pharmacokinetic limitations, restricted brain penetration, inter-individual variability, and a lack of robust GBM-specific clinical trials. These barriers reflect the broader challenges posed by GBM's biological complexity, metabolic adaptability, and resistance to therapeutic intervention. Compounds such as curcumin, resveratrol, epigallocatechin-3-gallate, omega-3 fatty acids, vitamin D, and melatonin modulate key oncogenic, inflammatory, and metabolic pathways and consistently enhance the effects of temozolomide and radiotherapy in experimental systems. Likewise, dietary patterns—including Mediterranean, plant-based, ketogenic, and fasting-mimicking approaches—influence systemic metabolism and inflammation, creating conditions that may be less permissive for tumor progression. However, these mechanistic and preclinical findings have not translated into evidence-based clinical benefit.

Clinical translation is limited by poor oral bioavailability, inadequate intratumoral exposure, heterogeneity in patient metabolism and tumor biology, and the absence of adequately powered, biomarker-driven trials. Importantly, the designation “natural” does not imply safety, and pharmacologic supplementation carries meaningful risks that warrant the same level of scrutiny as conventional therapies.

Progress in this field will require pharmaceutical-grade formulation, rigorous pharmacokinetic and mechanistic validation, biomarker-guided patient selection, and well-designed clinical trials with clinically meaningful and patient-centered endpoints. Until such evidence emerges, clinical practice should prioritize whole-food dietary patterns with established health benefits, correction of vitamin D deficiency, cautious use of low-risk adjuncts such as melatonin, and enrollment in clinical trials for investigational nutritional strategies. In a disease where even incremental advances are clinically meaningful, rigorous evaluation of dietary interventions offers an opportunity to define their proper role whether supportive, synergistic, or ultimately ineffective—and to guide patient care through evidence rather than assumption.

Looking forward, the most credible path is a shift from generic supplementation toward precision nutrition. This entails biomarker-guided patient selection - incorporating tumor metabolic subtype (from single-cell/spatial profiling or metabolic imaging), VDR and MGMT status, barrier integrity, and microbiome composition - so that each agent is tested in the population whose biology it is mechanistically suited to exploit. Priorities for the next generation of trials include pharmaceutical-grade, brain-penetrant or barrier-bypassing formulations with defined human pharmacokinetics; early-phase designs that confirm intratumoral target engagement through tissue or cerebrospinal fluid sampling and metabolic imaging; adaptive, biomarker-stratified randomization; rational combinations with temozolomide, radiotherapy, and emerging immunotherapies informed by the immunometabolic and myeloid mechanisms outlined above; and patient-centered endpoints including quality of life, neurocognition, and treatment tolerance. Framed this way, dietary and nutritional modulators are neither panaceas nor footnotes, but testable hypotheses whose rigorous evaluation - whether positive or negative—will meaningfully sharpen the standard of care in a disease where every incremental, reliably demonstrated gain matters.
